# Long-term computed tomography follow-up in great Danes with or without signs of osseous- associated cervical Spondylomyelopathy

**DOI:** 10.1186/s12917-019-1835-7

**Published:** 2019-03-12

**Authors:** Daniella Vansteenkiste, Paula Martin-Vaquero, Marília Bonelli, Luciana B. da Costa, Ronaldo C. da Costa

**Affiliations:** 1 College of Veterinary Medicine, The Ohio State University, 601 Vernon L. Tharp St, Columbus, OH USA; 2 Freelance medical and veterinary writer, Madrid, Spain

**Keywords:** Articular facet regularity and proliferation, Morphologic assessment, Morphometry, Natural history, Osseous-associated cervical spondylomyelopathy, Vertebral canal area

## Abstract

**Background:**

Osseous- associated cervical spondylomyelopathy (OA-CSM) has a high prevalence in Great Danes. In order to understand the progression of osseous changes, we aimed to perform a long-term computed tomographic (CT) follow-up study of Great Dane dogs with and without OA-CSM. Canine CSM is comparable to a common neurologic disease often diagnosed in older people termed cervical spondylotic myelopathy or degenerative cervical myelopathy, which is progressive in nature. The natural history of cervical spondylotic myelopathy in people has been well described, whereas there is scarce information on the natural history of canine OA-CSM. Our first goal was to evaluate if follow-up CT studies showed any changes compared to initial CT studies in Great Dane dogs with a diagnosis of OA-CSM. Our second goal was to establish whether clinically normal Great Danes went on to develop any vertebral changes or clinical signs consistent with OA-CSM. We enrolled Great Danes diagnosed with OA-CSM and clinically normal Great Danes who had previously participated in a prospective study. All dogs had clinical and CT follow-up evaluations.

**Results:**

Twelve Great Dane dogs were investigated: six OA-CSM affected and six clinically normal dogs. The median time between CT studies was 28 months (OA-CSM dogs) and 25 months (normal dogs). On follow-up CT, two OA-CSM-affected dogs developed new sites of stenosis, and two clinically normal dogs developed new sites of stenosis (one each). Disc spaces most commonly affected were C4-C5, C5-C6 and C6-C7. New sites of foraminal stenosis were noted in two of the CSM-affected and four of the clinically normal dogs. Morphometric evaluation showed no statistically significant differences between the initial and follow-up CT studies in the OA-CSM affected or normal groups.

**Conclusion:**

Our long-term CT follow-up study documented progression of vertebral canal stenosis in four out of twelve dogs. The majority of dogs did not develop new sites of stenosis or show progression of vertebral lesions.

## Background

Cervical spondylomyelopathy (CSM) is a common cause of cervical spinal cord dysfunction in giant and large breed dogs. CSM causes considerable gait abnormalities, leading to severe disability and pain. Two different forms of CSM have been documented, osseous-associated (OA-CSM), mainly seen in Great Danes and other giant breeds, and disc-associated (DA-CSM), where Dobermans and other large dogs are commonly affected. In OA-CSM, spinal cord and nerve root compression is caused by vertebral canal stenosis secondary to osseous proliferation of the vertebral arch, articular processes, and/or pedicles [1, 2]. Diagnosis of OA-CSM involves imaging evaluation of the cervical vertebral column. Basic radiography, myelography, computed tomography (CT), and magnetic resonance imaging (MRI) have all been described to aid in the diagnosis of OA-CSM, with each modality providing different information about the different anatomical structures [3–6]. It is generally accepted that MRI is the imaging modality of choice for dogs with suspected OA-CSM [7]. The MRI-based morphometry of the cervical vertebral column of clinically normal and OA-CSM affected Great Danes has been reported [2]. Vertebral canal stenosis secondary to osteoarthritic proliferation of the articular process or laminar/pedicular malformation and severe foraminal stenosis involving the cervical vertebral canal are characteristic of OA-CSM [8, 9]. Computed tomography has been compared to MRI and has shown its utility especially in OA-CSM [6]. Computed tomography was shown to be more consistent for evaluating cervical articular process joints [6].

Canine CSM is comparable to a common neurologic disease often diagnosed in older people termed cervical spondylotic myelopathy or degenerative cervical myelopathy, which is progressive in nature [10, 11]. The natural history of cervical spondylotic myelopathy in people has been well described [12]. Longitudinal studies including long-term imaging and clinical studies have played a key role in investigating and understanding the natural history of this condition in people.[12]Long-term longitudinal studies are paramount to understand the natural history of OA-CSM. Long-term follow-up information could help us identify factors associated with clinical deterioration and allow us to assess overtime how the condition evolves once treatment is initiated. There is a subset of human patients with CSM that are asymptomatic [13] and it is unknown whether this happens in Great Danes. Follow-up CT studies obtained in clinically normal dogs can help determine whether a similar situation exits in dogs with OA-CSM. Follow-up imaging studies have been performed in the Doberman breed investigating the progression of DA-CSM after medical and surgical treatments [14]. However, only one study has focused on the long-term vertebral changes in seven surgically treated giant breed dogs with cervical stenotic myelopathy, two of which were Great Danes with OA-CSM [14, 15].

The goal of the present study was two-fold: (1) to evaluate if follow-up CT studies showed any changes compared to initial CT studies in Great Dane dogs with a diagnosis of OA-CSM, and (2) to establish whether clinically normal Great Danes went on to develop any vertebral changes or clinical signs consistent with OA-CSM.

## Results

Twelve dogs were studied. Six were OA-CSM affected Great Danes and six clinically normal Great Danes. Of the six CSM-affected dogs, there were five castrated males and one spayed female. The median age at onset of clinical signs was 1.3 years (range: 0.7–3). The median age at the time of the first evaluation was 1.7 years (range: 1.3–4), and the median age at the second evaluation was 4.5 years (range: 4–7). The median follow up time was 2.3 years (range: 1.6–3). The median weight was 55.5 kg (range: 52–59). Of the six clinically normal dogs, two were male castrated and four were female spayed dogs. The median age at the time of the first evaluation was 2.2 years (range: 1.2–4), and the median age at the second evaluation was 4.5 years (range: 3–5.7). The median follow up time was 2.1 years (range: 1.6–2.75). The median weight was 50.1 kg (range: 46.5–75).

At the initial evaluation, physical exam findings in the OA-CSM affected dogs were normal except for one dog with a grade I-II/VI left systolic heart murmur that was deemed functional on electrocardiography, two dogs that were deaf, and one dog that suffered from glaucoma in the left eye. All OA-CSM affected dogs were ambulatory with proprioceptive ataxia upon initial examination. One dog was paraparetic and the remaining five dogs were tetraparetic. All OA-CSM affected Great Danes had some degree of conscious proprioception deficits. Mild neck pain was elicited in four out of six dogs at the time of the initial examination. Complete blood counts and serum biochemical panels were normal in all dogs. Initially, all OA-CSM affected dogs had been treated medically with a period of rest, plus a tapering course of prednisone in five dogs, and a non-steroidal anti-inflammatory (NSAID) drug in the remaining dog. One dog had had surgical decompression immediately after initial imaging. He had been managed medically for two months prior to initial CT.

On follow-up neurologic exam performed by a neurologist or a neurology resident (RdC and PMV) of the six OA-CSM affected dogs, all dogs continued to be ambulatory with proprioceptive ataxia. Two dogs were paraparetic and four dogs were tetraparetic. Three dogs developed supraspinatus and caudal thigh muscle atrophy. The neurologic exam (not sure we need to anything here. See comment) was unchanged in four dogs and improved in two (one of which had had surgery). Four dogs were no longer on any medication at the time of the follow-up examination, including the surgically treated dog, and two continued to receive anti-inflammatory doses of steroids.

All six clinically normal dogs had normal physical exams during the first evaluation, except for one dog that was deaf, and another dog who had had a tibial plateau leveling osteotomy two years earlier. All initial neurologic exams were within normal limits. No treatment had been performed on any of the normal dogs. On follow-up examination, one dog had a normal neurologic examination. The remaining five dogs had mild abnormalities on neurologic exam: two had a wide-based stance in the pelvic limbs, one had mild paraparesis, five had decreased conscious proprioception in the pelvic limbs, one had decreased conscious proprioception in the thoracic limbs, two had caudal cervical pain, and four dogs had thoracolumbar, lumbar or lumbosacral pain.

### Morphological assessment

**Fig. 1:**
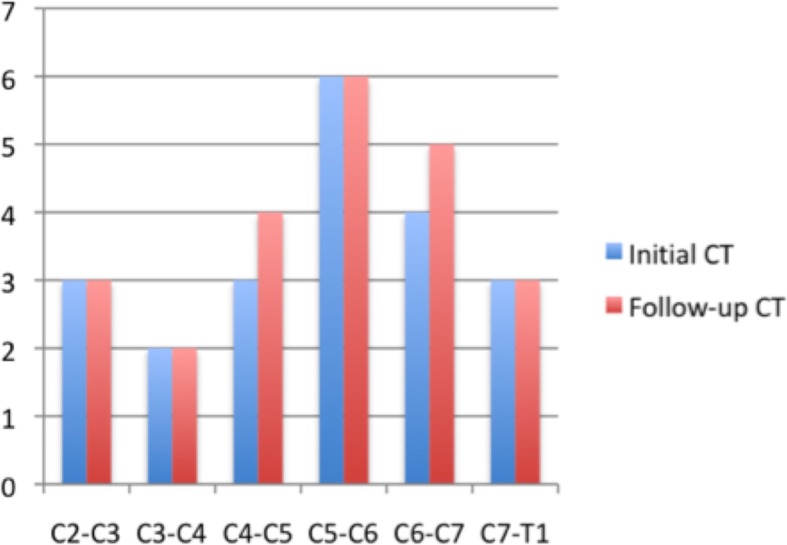
Number of stenotic sites identified at each intervertebral disc space in OA-CSM affected dogs on initial and follow-up CT scans

**Fig. 2:**
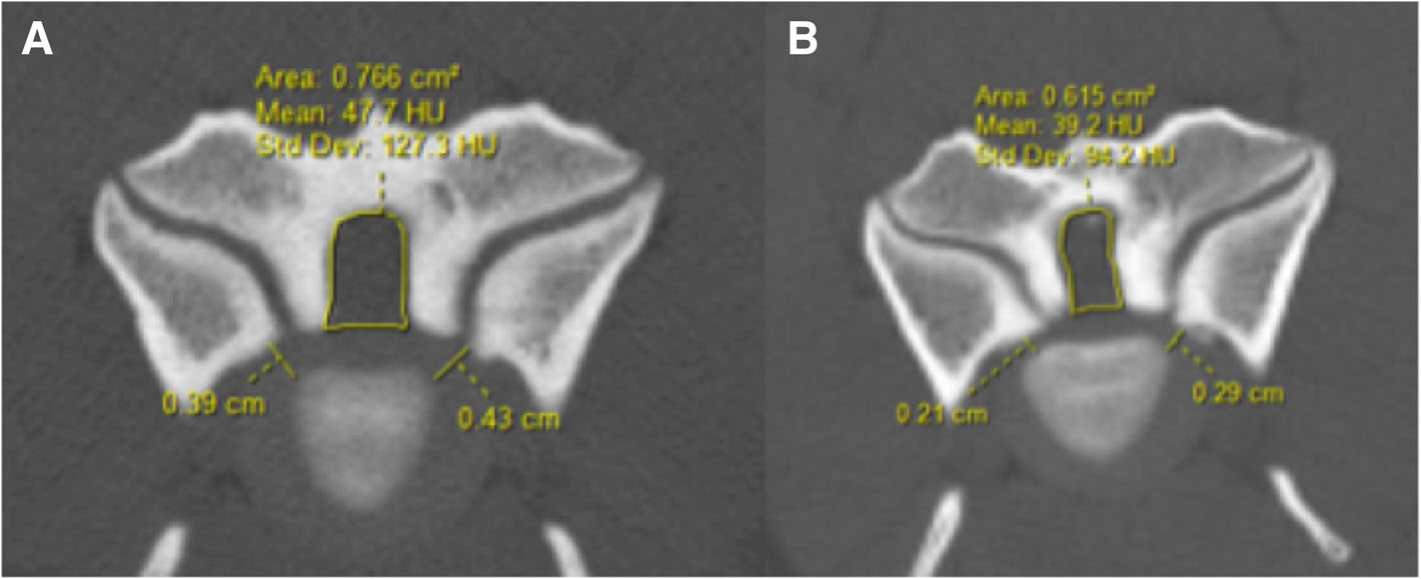
Computed tomography images of a 4 year-old Great Dane dog with OA-CSM. **a**) Initial and **b**) 31-month follow-up transverse images at the worst site of stenosis (C4-C5). The measurements shown were performed at each disc site for all dogs

**Fig. 3:**
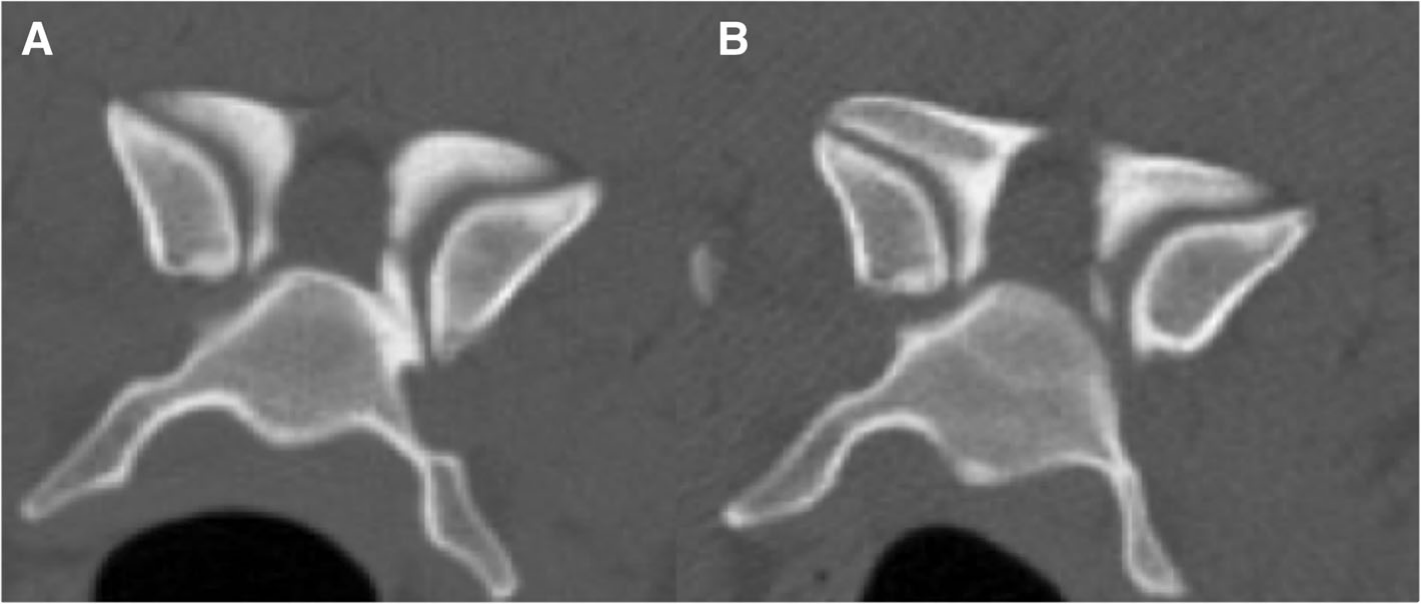
Computed tomography images of a 5.3-year-old Great Dane dog with OA-CSM. **a**) Initial and **b**) 24-month follow-up transverse image at the worst site of stenosis (C6-C7). There is no obvious progression in the degree of stenosis between the initial and follow-up CT studies

Forty-two disc spaces and a total of eighty-four articular facets were evaluated for both groups of dogs; the OA-CSM affected and clinically normal Great Danes. Multiple sites of stenosis were present in all CSM-affected dogs. The most common site of stenosis across the initial CT studies was C5-C6 (*n* = 6), followed by C6-C7 (*n* = 4). On follow-up CT, the number of stenotic sites located at C4-C5 and C6-C7 had increased by one each, whereas the remaining sites of stenosis were unchanged (Fig. 1). Twenty-one out of forty-two (21/42, 50%) intervertebral disc spaces were affected by some degree of vertebral canal stenosis on initial CT. Lateral stenosis was by far the most common type of stenosis observed, followed by dorsolateral and dorsal stenosis, with 15/21 (71%), 4/21 (20%), and 2/21 (9%) sites affected by each type of compression, respectively. On follow-up CT examination, 23/42 (55%) intervertebral disc spaces were affected by some degree of vertebral canal stenosis. Lateral stenosis remained most common followed by dorsolateral and dorsal stenosis, with 15/23 (65%), 5/23 (22%), and 3/23 (13%) sites affected by each type of compression, respectively. There were two new sites of stenosis, four sites of stenosis had worsened (Fig. 2), one had improved, and the remaining stenotic sites appeared unchanged (Fig. 3). The only site that showed improvement was observed in the dog that had undergone decompressive dorsal laminectomy; however, new bone formation was present around the laminectomy site.

In the clinically normal group, initial CT examination revealed two sites of dorsal stenosis in the same dog, located at C4-C5 and C5-C6; hence, only 2/42 (4%) of the sites evaluated showed stenosis among clinically normal dogs. On follow-up CT, 4/42 (9%) intervertebral spaces were affected by some degree of stenosis with two sites showing dorsal stenosis, one showing lateral stenosis, and one showing ventral stenosis. Compared to the initial CT evaluation, follow-up CT studies revealed two new sites of stenosis. The other two previously diagnosed stenotic sites remained unchanged. The most common site of vertebral canal stenosis was C6-C7 (*n* = 2). The dog with previous sites of stenosis at C4-C5 and C5-C6 had developed a new site of dorsal stenosis at C6-C7. This dog had also developed neurologic deficits characterized by mild ambulatory paraparesis, delayed proprioception in the pelvic limbs, and multifocal spinal pain (caudal cervical and lumbar pain). One dog with an initially normal CT developed ventral stenosis at C6-C7 with a mineralized disc visualized on CT. This dog developed delayed proprioception in the pelvic limbs and thoracolumbar pain.

On initial CT evaluation of the OA-CSM affected Great Danes, all dogs had at least one articular facet affected by either articular surface irregularities or articular facet proliferation, 59/84 (70%) articular processes had a normal articular surface, 17/84 (20%) were classified as grade 1, and 8/84 (10%) as grade 2. Thirty-eight out of eighty-four (45%) articular facets had no osseous proliferation, 42/84 (50%) were classified as grade 1, and 4/84 (5%) as grade 2. On follow-up exam, 50/84 (60%) articular processes had a normal articular surface, 27/84 (32%) were classified as grade 1, and 7/84 (8%) as grade 2. Thirty-four out of eighty-four (40%) had no osseous proliferation, 46/84 (55%) were classified as grade 1, and 4/84 (5%) as grade 2.

In the clinically normal group, all articular surfaces appeared normal and articular facet proliferation was present in 1/84 (1%) facets evaluated. On follow-up exam, 2/84 (2%) articular surfaces were classified as grade 1 in one dog. Two out of eighty-four (2%) articular facets were classified as grade 1 in terms of the osseous proliferation observed in another dog. The remaining articular surfaces and facets were within normal limits.

Foraminal stenosis was evaluated at all disc spaces for both left and right sides, with a total of 84 spaces evaluated in each group of dogs. In OA-CSM affected dogs, 5/6 dogs had evidence of foraminal stenosis at at least one disc space initially. All OA-CSM affected dogs had foraminal stenosis at follow up exam. Foraminal stenosis was initially present in 25/84 (30%) intervertebral disc spaces, whereas follow-up CT studies showed foraminal stenosis at 27/84 (32%) spaces. In the clinically normal dogs, foraminal stenosis was present in 2/84 (2%) spaces at initial exam versus 6/84 (7%) spaces on follow-up CT.

### Morphometric assessment

**Fig. 4:**
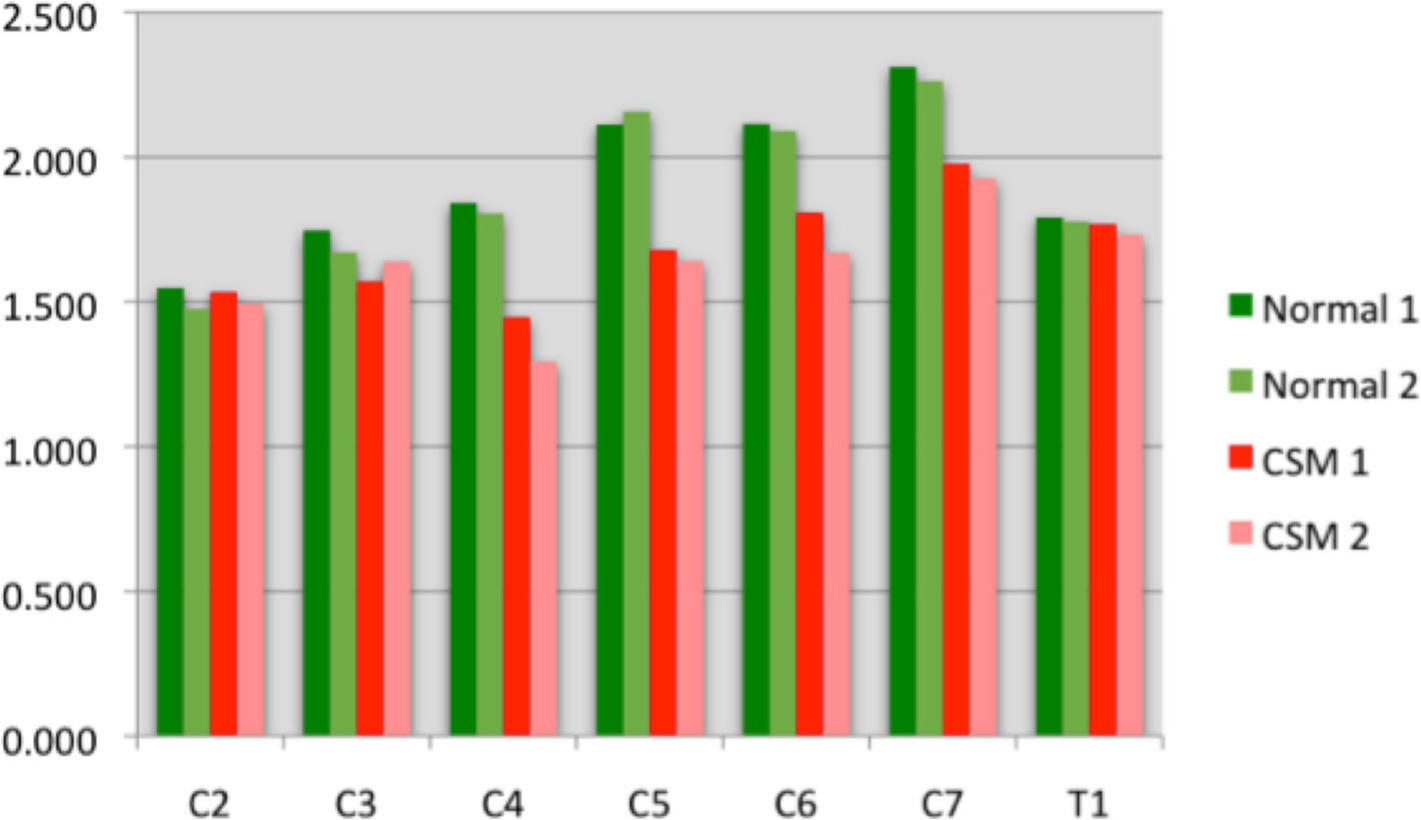
Average vertebral canal area (in cm2) at each disc space as measured on CT at initial (1) and follow-up (2) evaluation for clinically normal dogs and dogs with OA-CSM

A total of 840 measurements were performed in this study (35 measurements/dog/CT study). Three vertebral canal area measurements were performed for each disc space and the results are presented in Table 1. An example of some of the morphometric measurements are shown in Fig. 2. Mean vertebral canal measurements for each disc space are depicted in Fig. 4.

**Table 1:** Morphometric computed tomography (CT) results of the vertebral canal area in OA-CSM affected and clinically normal Great Danes (means ±95% confidence intervals) with *p*-value at the VCA_IVD_

Level	Dogs	VCA_cra_ Pre(cm2)	VCAI_VD_ Pre(cm2)	VCA_cau_ Pre(cm2)	VCA_cra_ Post(cm2)	VCA_IVD_ Post(cm2)	VCA_cau_ Post(cm2)	p-value at VCA_IVD_
C2-C3	OA-CSM	1.47 (±0.35)	1.51 (±0.32)	1.60 (±0.30)	1.51 (±0.23)	1.45 (±0.21)	1.70 (±0.28)	0.79
	Normal	1.72 (± 0.33)	1.60 (± 0.29)	1.77 (±0.33)	1.45 (± 0.26)	1.47 (±0.27)	1.74 (±0.32)	0.76
C3-C4	OA-CSM	1.47 (±0.21)	1.54 (±0.30)	1.57 (± 0.43)	1.43 (± 0.12)	1.60 (±0.21)	1.65 (±0.26)	0.75
	Normal	1.78 (± 0.33)	1.81 (±0.27)	1.95 (± 0.40)	1.59 (± 0.17)	1.70 (± 0.21)	2.06 (±0.32)	0.76
C4-C5	OA-CSM	1.31 (± 0.25)	1.44 (±0.34)	1.55 (± 0.40)	1.40 (±0.39)	1.32 (±0.39)	1.51 (± 0.40)	0.71
	Normal	1.87 (± 0.41)	1.94 (±0.28)	1.82 (±0.32)	1.76 (±0.25)	1.83 (±0.31)	1.99 (±0.22)	0.55
C5-C6	OA-CSM	1.63(±0.26)	1.67(±0.28)	1.60(±0.36)	1.60 (±0.24)	1.54 (±0.34)	1.66 (±0.40)	0.8
	Normal	2.16(±0.37)	2.12 (±0.30)	2.05(± 0.22)	2.05 (±0.33)	2.16(±0.29)	2.20(±0.38)	0.56
C6-C7	OA-CSM	1.90(±0.16)	1.80(±0.24)	1.81 (±0.14)	1.70 (±0.11)	1.65 (±0.20)	1.62 (±0.29)	0.66
	Normal	2.17 (±0.40)	2.13 (±0.46)	2.06 (±0.37)	2.07(±0.29)	2.10 (±0.43)	2.02(±0.27)	0.55
C7-T1	OA-CSM	1.81(±0.24)	1.95 (± 0.30)	2.12 (± 0.40)	1.78 (± 0.22)	1.97 (±0.29)	1.96 (±0.20)	0.78
	Normal	2.40 (±0.38)	2.34 (±0.36)	2.16 (±0.27)	2.21 (±0.42)	2.27 (±0.36)	2.15 (±0.30)	0.53
T1-T2	OA-CSM	1.85 (±0.21)	1.76 (±0.18)	1.84 (±0.21)	1.73 (±0.25)	1.74 (± 0.14)	1.80 (± 0.26)	0.77
	Normal	1.93 (±0.22)	1.81 (± 0.34)	1.87 (± 0.32)	1.87 (± 0.19)	1.77 (±0.31)	1.81 (± 0.27)	0.59

Morphometric evaluation showed no statistically significant differences between the initial and follow-up CT studies in the OA-CSM affected or normal groups. Table 1 shows the results of the morphometric vertebral canal area for all dogs and the *p*-values for the VCA_IVD_ measurements.

Intraclass correlation of single measures for all intra- and interobserver vertebral canal area measurements were 0.958 and 0.908, respectively, indicating excellent intra-and interobserver agreement. Foraminal heights were analyzed separately and the intra- and interobserver correlations were 0.888 and 0.559, respectively, indicating excellent intraobserver agreement and fair interobserver agreement.

## Discussion

In this study we documented the long-term progression of vertebral changes in Great Dane dogs with and without OA-CSM using CT imaging. Overall, we noticed progression of vertebral lesions during the 28 month follow-up period in 4/12 dogs evaluated. The remainder of the dogs did not develop new lesions. The morphologic assessment was paired with a morphometric analysis to offer a more objective evaluation. No significant differences were seen across the morphometric data between initial and follow-up CT studies, supporting the morphologic findings that showed no obvious progression of lesions in the majority of dogs during the study period.

Clinical signs of OA-CSM appeared to progress slowly, with all dogs except one being unchanged to improved in their clinical status at follow-up, regardless of changes observed on CT. Medical management improved or stabilized the neurologic exam of all dogs except for one dog, which was then treated surgically. In Dobermans, DA-CSM presents later in life and a median survival time of 36 months has been reported for medical and/or surgical treatment [14]. With OA-CSM presenting much earlier in life for the giant breeds, the disease has more time to progress. There has been one follow-up study in surgically treated giant breeds with OA-CSM that included short and long term imaging follow-up [15]. In that study, all seven dogs underwent distraction and stabilization with PMMA and/or bone grafts. They were followed for up to 4 years, which included a neurologic exam and follow up CT imaging in four dogs. Recurrence of neurological signs was common [15]. In our study, the follow-up neurologic exam of the OA-CSM affected dogs remained unchanged or improved in all six dogs, including five dogs that had been medically managed.

All OA-CSM affected dogs had multiple sites of stenosis on both CT evaluations. Two of the clinically normal dogs developed new sites of stenosis at follow-up with one dog remaining clinically normal, whereas the other one became clinical for OA-CSM. Neither one of these two dogs required treatment. One of these dogs also had evidence of mineralized disc material within the vertebral canal on follow-up CT. This study confirmed that OA- CSM affected dogs, even after being medically or surgically managed, can develop new morphologic changes associated with OA-CSM overtime without major deterioration in their clinical signs. Also, our study showed that clinically normal Great Danes can eventually develop morphologic changes consistent with OA-CSM, sometimes accompanied by some degree of neurological signs consistent with cervical disease, but also with no obvious signs of cervical disease present, or even developing neurological abnormalities unrelated to a cervical myelopathy, such as multifocal spinal pain. Owners of the clinically normal dogs enrolled in this study had rarely perceived any changes in their dogs’ ability to ambulate or perform daily activities.

In a study performed in humans, 57% of a randomly selected population had nonmyelopathic spondylotic cervical cord compression (NMSCCC) [13]. This is also known as asymptomatic CSM, where cervical cord impingement or compression is found without any clinical signs. They also found an increase in the percentage of cervical spinal cord compression associated with age, which might be an explanation as to why some of the clinically normal dogs developed imaging findings consistent with OA-CSM on follow-up CT without any significant clinical signs [13]. In the aforementioned study, it was found that the highest capacity to predict NMSCCC was using an anteroposterior diameter of the cervical vertebral canal and the spinal cord compression ratio on MRI, [13] which we were unable to calculate using CT images. This is an interesting concept used in human medicine that has not been investigated in veterinary medicine. Based on this concept, some of our clinically normal dogs might have actually had asymptomatic OA-CSM. Vertebral canal changes have been reported in 1 clinically normal Great Dane however it is unknown if these asymptomatic cases truly exist in canine OA-CSM. [2] The small sample size in this study precludes any clear conclusion. Vertebral changes in clinically normal horses have been reported [16]. A study found abnormal articular processes in clinically normal horses on cervical spinal radiographs; however, the clinically affected horses showed more severe bony malformations than the control group [16]. Future MRI studies may also be useful for investigating ratios such as those described in people in dogs with OA-CSM [13].

There is some evidence that shows that DA-CSM appears to be inherited as an autosomal dominant trait in the Doberman [17]. A genetic component of the disease is also suspected in Great Danes due to an early onset of disease and higher incidence in some lineages. Normal cervical spine imaging is required for appropriate phenotyping. Determining a genetic component to this disease is complicated due to the lack of association between the progression of clinical signs and imaging features of the disease. Growth rate disparities have been demonstrated in Great Dane rib growth plates, where they had larger absolute proliferative and hypertrophic zones compared to miniature poodles [18]. That study found that disparities in longitudinal growth were explained by endocrinological differences, upregulation of the vitamin D pathway, and differences in the parathyroid hormone-related peptide during rapid growth rates [18]. Comparing the endocrinological profiles of clinically normal and OA-CSM affected Great Danes could be interesting to try to identify if those dogs prone to developing hypertrophic and proliferative compressive lesions at a younger age might show calcium metabolism profiles different to dogs with no vertebral lesions or dogs that develop lesions later in life.

In this study, we reported a lower percentage of disc spaces affected by some degree of vertebral canal stenosis compared with previous studies; [7] however, previous data was drawn from MRI studies and not from CT. This may also be due to a smaller number of cases that all responded favorably to medical or surgical management. In our study, lateral stenosis of the vertebral canal was by far the most common cause of stenosis followed by dorsolateral stenosis. This is in agreement with other studies published [7, 8]. However, the degree of dorsal compression observed in our study appeared less marked compared to what has been previously described by Gutierrez et al., [7] which could be explained due to the greater soft tissue resolution obtained with MRI [7, 19].

An interesting aspect of the study is that five out of six initially clinically normal dogs went on to developing neurologic deficits by the time the follow-up CT examination was performed. Cervical stenotic sites were only documented in two of these dogs, so the neurological signs observed in the remainder three dogs probably had other underlying causes; however, the fact that two initially clinically normal dogs went on to develop stenotic sites complicates the comparison between OA-CSM affected Great Danes and clinically normal dogs however we believe this is important information and highlights the importance of performing longitudinal clinical and imaging studies in normal and abnormal populations to understand how disease develops and progresses.

Limitations of the study include that imaging follow-up was only performed using CT and not MRI, which prevented us from assessing the spinal cord parenchyma. Also, the sample size was small and a potential selection bias exists as our population of dogs had to be available for long term follow up and imaging. The response to treatment may play a role in the progression of CT findings as the dogs included responded favorably to medical or surgical management. Our original intention was to include a larger group of dogs from those who had been enrolled in the original prospective study, but some dogs were deceased and some owners could not be reached. Another possible limitation of this study is the inclusion of a surgically treated OA-CSM affected dog. We included this dog due to our small sample size and because little information is known regarding remodeling of the vertebral canal after surgery. The vertebral canal could be measured on the follow up CT.

Morphometric evaluations did not show statistically significant differences either between initial and follow-up CT studies or between normal and OA-CSM-affected dogs. This was likely due to the small sample size and wide variability in the measurements across different dogs. The morphologic assessment might be more clinically relevant and provide an easier way to evaluate if progression of the lesions has happened, however, morphologic evaluations also carry increased subjectivity.

Intra- and interobserver correlations were excellent apart from the foraminal heights, for which interobserver agreement was fair. This is most likely due to the subjectivity of this measurement on CT. This measurement was performed at the level of the intervertebral disc where the soft tissue is not as well defined on CT images as compared to MRI, making it more challenging to determine the exact foraminal heights. Different imaging windows and the localizer tool were used to minimize this variation; however, MRI appears to be a more accurate imaging method when measuring foraminal heights [8]. There were no significant differences in the number of sites with foraminal stenosis recorded on the morphologic assessment when comparing initial and follow-up studies so, although foraminal stenosis is a common feature in OA-CSM affected Great Danes, [9] it does not appear to play a major role in the progression of the disease.

Further studies will be required to evaluate this disease process as the Great Danes continue to age. MRI follow-up studies would be useful to evaluate soft tissue compression, intraparenchymal changes, and spinal cord atrophy over time.

## Conclusion

In conclusion, our long-term CT follow-up study documented progression of vertebral canal stenosis in 4/12 dogs evaluated. The majority of dogs did not develop new sites or show progression of vertebral lesions. It is possible that the response to treatment plays a role in the progression of CT findings. These findings should be taken into consideration in the decision-making process of the management of dogs with OA-CSM.

## Methods

Great Danes with a confirmed diagnosis of CSM and clinically normal Great Danes from a previous prospective study at the Ohio State University [2, 6, 8] were enrolled on this long-term CT follow-up study. Inclusion criteria were participation in the previous study, MRI and CT imaging on initial evaluation, and follow-up CT imaging performed in 2014. CSM-affected Great Danes had to have a prior diagnosis of CSM based on clinical assessment and MRI. The clinically normal group of Great Danes was required to have a normal initial neurologic exam. All dogs enrolled in this follow-up study had undergone complete physical and neurologic examinations performed by two of the investigators (PMV and RdC). The initial evaluation and imaging were performed between April 2011 and September 2012. Repeat evaluation and imaging were performed in April 2014. The study was performed in accordance with the guidelines and approval of the Clinical Research Advisory Committee and the Institutional Animal Care and Use Committee. Written owner consent was obtained prior to each follow-up evaluation and imaging. A complete blood count and serum biochemical panel were performed prior to undergoing sedation for CT of the cervical spine. If a heart murmur was auscultated, evaluation by a cardiologist was pursued and electro- and echocardiography were performed as necessary. Computed tomography of the cervical vertebral canal involved imaging of C2-T3. All CT examinations were done with dogs under sedation using an 8-slice CT scanner (GE LightSpeed Ultra 8-slice, GE Healthcare, Waukesha, WI). Each dog was sedated with hydromorphone (0.05–0.1 mg/kg IV, West- Ward Pharmaceuticals, Eatontown, NJ) and dexmedetomidine (4–8 mcg/kg IV, Dexdomitor®, Pfizer Animal Health, New York, NY). Dogs were positioned in sternal recumbency with the head and neck extended in neutral position. Scanning parameters included: 120 kV, automatic mA (min = 100 mA), and axial mode, 2.5 mm contiguous transverse slices using standard and bone algorithms. Transverse slices were obtained parallel to the vertebral endplates.

All CSM-affected dogs were initially treated with medical management. One dog was later treated surgically. Medical management consisted of prednisone at a starting dose of 0.5–1 mg/kg/day. The medication was initially started twice a day and then was gradually tapered to every 24 and 48 h after 10 days at each dose. The lowest dose of prednisone that was relieving clinical signs was maintained in those CSM-affected dogs that could not be tapered off of the prednisone. Non-steroidal anti-inflammatories were also used in some cases, but never in conjunction with prednisone. All dogs had neurologic exams performed at rechecks as needed. Surgical decompression for the only affected dog that was surgically treated involved a dorsal laminectomy over the main sites of stenosis (C3-C4 and C4-C5). A standard surgical approach was performed, and the spinal cord appeared visibly decompressed prior to closure. No implants or grafts were used during this procedure. Exercise restriction was also instituted for four weeks in all affected dogs. The use of a harness instead of a neck collar was recommended. Short leash walks were allowed, but free running and playing was discouraged. All canine patients were discharged to their owners after diagnostic imaging and were re-evaluated as deemed necessary.

CT images were reviewed by two observers (DV and MB). The observers were not blinded to the clinical status of the dogs. The images were independently reviewed, and morphologic and morphometric assessments were performed using dedicated software for medical imaging analysis (ClearCanvas Workstation). First, the initial CT was evaluated for all dogs, CSM-affected and clinically normal dogs, in random order. The follow-up CT study was then reviewed one to three weeks after the initial CT had been assessed. The observers did not have the results of the previous examination and were not blinded as to the clinical status of the dogs. The images were evaluated for morphologic features, specifically, number and sites of stenosis, direction of stenosis, site of the most compressive lesion, regularity of the articular surface, presence of subchondral bone sclerosis, degree of articular proliferation, and foraminal stenosis. The grading system was adapted for CT from previously published techniques [6, 7, 20]. Each articular surface was evaluated for the regularity of the articular surface (0 = normal, 1 = smooth, but with osteochondral sclerosis, and 2 = irregular with sclerosis), and the degree of osseous proliferation (0 = none, 1 = < 25%, and 2 = 25–50% increase in size). The follow-up CT was also evaluated for new sites of stenosis. Once all evaluations were completed, the sites showing stenosis were evaluated side by side by pairing up the initial and follow-up CT studies at the same time. The sites of stenosis were subjectively graded as static, worsened, or improved.

Morphometric measurements were performed separately for both CTs on the transverse images: vertebral canal area was measured at the caudal endplate of the cranial cervical vertebrae, at the disc space, and at the cranial edge of the caudal endplate (VCA_cra_, VCA_ivd_, VCA_cau_). The localizer function on ClearCanvas was used to help determine the limits of the vertebral canal area. Left and right foraminal heights were measured on the transverse images at the level of the intervertebral disc space. Measurements were obtained from C2-C3 through T1-T2.

To assess intra- and interobserver agreement, all measurements were repeated in four dogs (two CSM-affected and two clinically normal dogs) that were randomly selected using a random number generator. Measurements were repeated at least one month after the initial measurements had been obtained by the same investigator (DV) who had performed the first round of morphologic and morphometric assessments. The second investigator (MB) performed measurements for the inter-observer agreement one month after the first investigator.

Intra- and interobserver agreements were tested with a two-way mixed intraclass correlation coefficient (rho), where repeated measurements were compared and the average measurement value was reported. When the correlation coefficient is close to 1, agreement is considered excellent, whereas a correlation coefficient close to 0 indicates lack of agreement.

All data were assessed for normal distribution using the Shapiro-Wilk test. Vertebral canal area measurements were evaluated with a repeated measures analysis in the MIXED procedure of Statistical analysis software (SAS) version 9.4 (Statistical Analysis System, SAS Institute, Cary, North Carolina), with the assessed dogs and region of the vertebral column considered as random. The compound symmetry covariance structure was used to account for non-independence of observations within the same dog. Differences were considered significant by use of a Fisher’s exact test at a value of *P* < 0.05.

## Abbreviations

cau: Caudal
cra: Cranial
CSM: Cervical spondylomyelopathy
CT: Computed Tomography
DA-CSM: Disc-associated cervical spondylomyelopathy
IVD: Intervertebral disc
MRI: Magnetic Resonance Imaging
NMSCC: Nonmyelopathic spondylotic cervical cord compression
OA-CSM: Osseous-associated cervical spondylomyelopathy
SAS: Statistical Analysis Software
VCA: Vertebral canal area
